# Altered Hippocampal Neurogenesis and Amygdalar Neuronal Activity in Adult Mice with Repeated Experience of Aggression

**DOI:** 10.3389/fnins.2015.00443

**Published:** 2015-12-01

**Authors:** Dmitry A. Smagin, June-Hee Park, Tatyana V. Michurina, Natalia Peunova, Zachary Glass, Kasim Sayed, Natalya P. Bondar, Irina N. Kovalenko, Natalia N. Kudryavtseva, Grigori Enikolopov

**Affiliations:** ^1^Institute of Cytology and Genetics, Siberian Branch of Russian Academy of SciencesNovosibirsk, Russia; ^2^Department of Nano-, Bio-, Information Technology and Cognitive Science, Moscow Institute of Physics and TechnologyMoscow, Russia; ^3^Cold Spring Harbor Laboratory, Cold Spring HarborNY, USA; ^4^Department of Anesthesiology, Stony Brook School of MedicineStony Brook, NY, USA; ^5^Center for Developmental Genetics, Stony Brook UniversityStony Brook, NY, USA

**Keywords:** adult neurogenesis, aggression, hippocampus, amygdala, social conflict, c-fos-positive cells, anxiety, autism

## Abstract

Repeated experience of winning in a social conflict setting elevates levels of aggression and may lead to violent behavioral patterns. Here, we use a paradigm of repeated aggression and fighting deprivation to examine changes in behavior, neurogenesis, and neuronal activity in mice with positive fighting experience. We show that for males, repeated positive fighting experience induces persistent demonstration of aggression and stereotypic behaviors in daily agonistic interactions, enhances aggressive motivation, and elevates levels of anxiety. When winning males are deprived of opportunities to engage in further fights, they demonstrate increased levels of aggressiveness. Positive fighting experience results in increased levels of progenitor cell proliferation and production of young neurons in the hippocampus. This increase is not diminished after a fighting deprivation period. Furthermore, repeated winning experience decreases the number of activated (c-fos-positive) cells in the basolateral amygdala and increases the number of activated cells in the hippocampus; a subsequent no-fight period restores the number of c-fos-positive cells. Our results indicate that extended positive fighting experience in a social conflict heightens aggression, increases proliferation of neuronal progenitors and production of young neurons in the hippocampus, and decreases neuronal activity in the amygdala; these changes can be modified by depriving the winners of the opportunity for further fights.

## Introduction

Aggressive behavior helps to ensure survival, provides advantage in competition, and communicates social status (Scott, 1971). In the situation of a social conflict, for both animals and humans, positive fighting experience may be rewarding and further positive reinforcement may increase the propensity for aggressive behavior (Scott, 1971; Baron and Richardson, 1994; Fish et al., 2002; Takahashi and Miczek, 2014). Male rodents with a prior record of winning in a social conflict setting attack more frequently and may develop violent behavior patterns with little regard to the submissive signals of the opponent or to an unfamiliar environment (Van de Poll et al., 1982; Kudryavtseva et al., 2004; Kudryavtseva, 2006; Natarajan et al., 2009; Natarajan and Caramaschi, 2010). For instance, mice that have won a series of fights in a social conflict paradigm display aggressive and hostile behavior (attacks, threats, and indirect aggression) even toward a much heavier and stronger male, a juvenile, a female, or a defeated conspecific demonstrating overt signs of submissiveness (Kudryavtseva, 2006). Furthermore, mice with winning experience in a prolonged series of fights develop psychopathological behavioral traits, manifested as abnormal aggression, heightened hostility, pronounced anxiety, stereotypic behaviors, and disturbances in motivated behavior (Kudryavtseva, 2006; Caramaschi et al., 2008). Remarkably, if winner animals are denied opportunities to engage in further fights for 2–3 weeks, they demonstrate a level of aggression higher than before deprivation (Kudryavtseva, 2006; Kudryavtseva et al., 2011).

Limbic regions such as amygdala and hippocampus are involved in innate social behaviors and response to social stress; they have also been implicated in aggressive behavior (Comai et al., 2012; Hong et al., 2014; Rosell and Siever, 2015). Indeed, amygdala and hippocampus are critical for the emotional and cognitive functions such as social recognition, fighting, mating, fear, or motivated behaviors (Lopez et al., 2004) that are affected in animals with prolonged positive fighting experience (Kudryavtseva, 2006). Fighting-evoked changes in the hippocampus, besides altered neuronal activity and synaptic plasticity, may involve division of neural stem and progenitor cells and production of new neurons in the dentate gyrus (DG; Lagace et al., 2010; Samuels and Hen, 2011; Aimone et al., 2014; Cameron and Glover, 2015). Notably, mice that have been genetically selected for high levels of aggression demonstrate a higher basal level of cell division in the DG than mice selected for low aggression (Veenema et al., 2004, 2007). Furthermore, in rats housed in a visible burrow system, social dominance affects hippocampal neurogenesis, with more new neurons observed in the DG of the dominant males in comparison with subordinates or controls (Kozorovitskiy and Gould, 2004).

Here, we investigated whether continuous positive fighting experience in a mouse model of social conflict affects hippocampal neurogenesis and neuronal activity in the hippocampus and the amygdala. We also examined whether division of neuronal progenitors and neuronal activity are further altered by fighting deprivation, since persistence of the behavioral and cellular changes after cessation of agonistic interaction may mark psycho- or neuropathological changes in the affected animals. Our results indicate that a prolonged series of positive fighting episodes augments production of new neurons in the DG and alters activation of neurons in the hippocampus and the basolateral amygdala.

## Materials and methods

### Animals

Adult male mice from four different lines were used as experimental subjects: wild type C57BL/6J mice obtained from Jackson Laboratories; wild type C57BL/Icg mice from a C57BL/6J-derived stock maintained at the animal facility of the Institute of Cytology and Genetics, SD RAS (Novosibirsk, Russia); transgenic Nestin-GFP homozygous mice (Nestin-GFP), previously backcrossed to C57BL/6J for over 10 generations (Mignone et al., 2004); and transgenic Nestin-GFP heterozygous mice (Nestin-GFPhet), obtained as a cross between homozygous Nestin-GFP and C57BL/6J mice. Animals were housed under standard conditions (12:12 h light/dark regime starting at 8:00 am, at a constant temperature of 22 ± 2°C, with food in pellets and water available *ad libitum*). Mice were weaned at 3 weeks of age and housed in groups in standard plastic cages. Experiments were performed with 10–12 week old animals. All behavioral experiments were performed by the same experimenters. All procedures were carried out in compliance with the Guide for the Use and Treatment of Laboratory Animals from the National Institutes of Health and with the European Communities Council Directive (86/609/EEC). Animal procedures were approved by the Institutional Care and Use of Laboratory Animals (IACUC) committees of Cold Spring Harbor Laboratory and the Institute of Cytology and Genetics.

### Generation of positive fighting experience in male mice

Prolonged negative and positive social experience (defeats and victories) in male mice was induced by daily agonistic interactions under chronic social conflict as detailed in Kudryavtseva et al. (2014). In brief, pairs of weight-matched animals were placed in a cage (14 × 28 × 10 cm) bisected by a perforated transparent partition allowing the animals to see, hear, and smell each other, but preventing their physical contact. The animals were left undisturbed for 2 or 3 days to adapt to new housing conditions and sensory environment before exposing them to direct interactions. Every afternoon (2–5 pm) the cage lid was replaced with a transparent one and 5 min later (a period for bringing individual animals into the same alert state), the partition was removed for 10 min to encourage agonistic interactions. While both males displayed aggressiveness upon the first interaction, within two or three encounters with the same opponent the dominance of one of the males was firmly established. The winning male would be attacking, biting, and chasing another male, who would be displaying only defensive behavior (sideways postures, upright postures, withdrawal, lying on the back or freezing). Aggressive confrontations between animals were discontinued by lowering the partition if a strong display of aggressive behavior has lasted 3 min (sometimes less, to avoid wounding of the defeated male). Each winning mouse remained in its original cage and its behavior in agonistic interactions and partition tests (below) was video recorded for 10 min during its last encounter. Each loosing male was exposed to the same winner for 3 days, and afterwards each day, following the fight, placed in an unfamiliar cage with an unfamiliar winner behind the partition. This procedure was performed once a day for 21 days and yielded an equal number of winners and defeated mice.

Following groups of animals were analyzed for their behavior, progenitor cell division, production of new neurons, or c-fos activity: winners—males with a consistent winning experience during 10 (Win10) or 21 (Win21) days of agonistic interactions; fighting-deprived winners (Win21-D)—winner males subjected, after 21 days of agonistic interactions, to a no-fight period for 2 weeks; and controls—males without consecutive experience of agonistic interactions.

Percentage of male mice engaged in fighting was different for different strains. Ninety to Ninety-five percent of C57BL/Icg mice, 70% of C57BL/6J mice, and 67% of Nestin-GFP and Nestin-GFPhet mice displayed aggression in agonistic interactions during the 21-day period. These variations may be due to minor genetic differences between strains, but may also reflect minor modifications of experimental conditions at different animal facilities.

To examine the effects of fight deprivation, C57BL/Icg males that emerged as winners after 21 days of agonistic interactions (Win21) were placed in separate cages with a defeated male behind the partition and subjected to a no-fight period of 2 weeks (Win21-D; Kudryavtseva et al., 2011). After the no-fight period and 1 day before testing, for each winner the defeated mouse in the cage was replaced by an unfamiliar defeated mouse. Behavioral activity was video recorded to follow the effects of fighting deprivation on aggressive and associated forms of behavior. No significant differences in any of the examined behavioral parameters were found between the animals of the Win21 and Win21-D groups after 21 days of agonistic interactions.

### Agonistic interaction test

The following behavioral domains were recorded for 10 min for the aggressor animals: (1) *Attacks* (attacking, biting and chasing); (2) *Aggressive grooming* (mounting the defeated animal's back, holding it down, and spending much time licking and nibbling at the scruff of the defeated male's neck; during this time, the defeated mouse remains wholly immobilized or, sometimes, stretches out its neck and again freezes under the aggressor animal); (3) *Digging* (digging up and scattering the sawdust on the defeated animal's territory such as kick-digs: pulling the sawdust forwards with the forepaws; and push-digs: pushing the sawdust backwards with the hind paws); (4) *Hostile behavior* (the total time spent attacking, aggressively grooming and digging); (5) *Threats* (number of tail rattling); (6) *Rotations* (quick 180° turns with jumps).

The following variables were measured: (a) latency to the first attack, s; (b) number (for the behavioral domains 1, 3, 5, 6 above); (c) total time, s (for the domains 1, 2, 3, 4); (d) percentage of males demonstrating a particular behavior (for the domains 2, 5, 6).

If an animal did not display any of the behaviors listed above, the latency to these events was recorded as 600 s (test duration) and all other variables were recorded as zero. The total time of attacks in comparison with the last test of agonistic interactions of the same male was used to define the animals as those in which aggression level has increased after the fighting deprivation, with a difference of 10–15 s in attacking time used as a criterion for such increase (in those rare cases when this parameter did not differ significantly before and after deprivation, the number and the latency of attacks, and total time of hostile behavior were also taken into consideration). Separate groups of fighting-deprived males were used for the biochemical and behavioral studies.

### Partition test

Partition test was employed as a tool for estimating behavioral reactivity of mice to a conspecific placed behind a transparent perforated partition dividing the experimental cage into equal parts (Kudryavtseva et al., 2014). The number of approaches to the partition and the total time spent near it (moving near the partition, smelling and touching it with one or two paws, clutching and hanging, putting the nose into the holes, or gnawing the holes) were scored for 5 min and used as indices of reacting to a familiar or an unfamiliar partner. This test measures level of aggressive motivation, with behavioral activity near the partition in reaction to the partner in the neighboring compartment (before the onset of agonistic interactions) correlating with expression of aggressiveness (attacking behavior) in the agonistic interactions that follows the removal of the partition (Kudryavtseva, 2003).

### Elevated plus-maze test

The elevated plus-maze test was conducted using a maze consisting of two open and two closed arms as described (Kovalenko et al., 2014). Elevated plus-maze consisted of two open arms (25 × 5 cm) and two closed arms (25 × 5 × 15 cm), with two arms of each type opposite to each other and extending from a central platform (5 × 5 cm). The maze was placed in a dimly lit room and the following behavioral parameters were recorded during 5 min: (1) open arm entries (four paws in the open arm), closed arm entries (four paws in the closed arm), and central platform entries; (2) total entries; (3) time spent in the open arms, closed arms, and central platform; (4) the number of passages from one closed arm to another; (5) the number of head-dips (looking down on the floor below the plus-maze); (6) the number of peepings when staying in closed arms (extending the head from the closed arm and quickly pulling back). The entries into the closed and open arms and the central platform were determined as percentages of the total entries, and time spent in the closed and open arms and the central platform was determined as percentages of the total testing time. The maze was thoroughly cleaned between sessions.

### Thymidine analog labeling, transcardial perfusion, and tissue sectioning

Procedures were performed following published protocols (Encinas and Enikolopov, 2008; Park and Enikolopov, 2010; Encinas et al., 2011a). Briefly, animals were injected with 5-bromo-2′-deoxyuridine (BrdU; 150 mg/kg) and 2 h later animals were deeply anesthetized with 3% Avertin (2,2,2-tribromoethanol, Sigma-Aldrich, St. Louis, MO) and subjected to transcardial perfusion with 30 ml of phosphate-buffered saline (PBS) followed by 30 ml of 4% paraformaldehyde (PF) in PBS, pH 7.4. The brains were removed and post-fixed overnight with the same fixative at 4°C, then transferred to PBS with 0.1% sodium azide and kept at 4°C until sectioning. Before sectioning, the brains were cut sagittally into two hemispheres. Brain hemispheres were randomly selected for each animal and serial 50 μm-thick sagittal (for analyzing the DG) or coronal (for analyzing the amygdala) sections of the hemispheres were collected using Vibratome 1500 (Vibratome, St. Louis, MO). Each sixth section was analyzed (i.e., subsets of sections at 300 μm intervals were used for immunocytochemistry and analysis). For each series of experiments, brain sections from all experimental groups were processed simultaneously throughout all stages of the immunohistochemical procedures.

### Immunocytochemistry, image capture, and analysis

Immunostaining was carried out following standard protocols, as described previously (Encinas and Enikolopov, 2008; Encinas et al., 2011a,b; Park et al., 2013). Briefly, brain sections were incubated with the blocking and permeabilization solution (PBS containing 1% Triton-100X and 3% goat serum) for 2 h at room temperature (for BrdU detection sections were first denatured in 2 N HCl at 37°C for 1 h and neutralized by 0.1 M borate) and then incubated overnight at 4°C with the primary antibodies: rat anti-BrdU (1:300, BU1/75, Accurate Chemical Inc., Westbury, NY), chicken anti-GFP (1:400 dilution, GFP-1020, Aves Labs, Tigard, OR), rabbit anti-glial fibrillary acidic protein (GFAP; 1:500 dilution, Z-0034, DakoCytomation, Carpinteria, CA), chicken anti-doublecortin (DCX; 1:500 dilution, DCX, Aves Labs), or rabbit anti-c-fos (1:300, sc-253, Santa Cruz Biotechnology, Santa Cruz, CA), with primary antibody diluted in PBS containing 0.2% Triton-100X and 3% goat serum. After washing with PBS, the sections were incubated with fluorochrome-conjugated AlexaFluor 488 goat anti-rat secondary antibodies (1:400, Molecular Probes, Eugene, OR), AlexaFluor 568 goat anti-rabbit secondary antibodies (1:400, Molecular Probes), AlexaFluor 488 goat anti-rabbit secondary antibodies (1:400, Molecular Probes), Cy5 goat anti-chicken (Jackson Immunoresearch, West Grove, PA), diluted in PBS containing 0.2% Triton-100X and 3% goat serum for 2 h at room temperature. After washing with PBS, the sections were mounted on gelatin-coated slides with DakoCytomation Fluorescent Mounting Medium (DakoCytomation).

For counting BrdU-positive cells, 8–9 sagittal DG-containing sections per mouse were analyzed (the set of sections, at 300 μm intervals, covering the entire dorsal and ventral DG). For counting c-fos-positive cells, 3–4 coronal sections covering the key amygdala nuclei (bregma –2.255 to –1.355 mm) were analyzed for each mouse. Images were collected using a PerkinElmer UltraView spinning disk fluorescent microscope (PerkinElmer, Wellesley, MA). The DG and amygdala were identified and anatomically demarcated in accordance with the stereotaxic mouse brain atlas (Lein et al., 2007; Franklin and Paxinos, 2008; http://mouse.brain-map.org). Three subregions were identified in the amygdala: the lateral and basal nuclei summarized as the basolateral region (BLA); basomedial nucleus (BMA); and the central nucleus (CeA). Quantitative analysis of cell populations was performed by means of design-based (assumption-free) stereology (Encinas and Enikolopov, 2008), aided, when necessary, by Volocity v.6.0 software (PerkinElmer).

For phenotyping stem and progenitor cells sections were analyzed by confocal microscopy for the presence of BrdU-labeled radial glia-like quiescent neural progenitors (QNP) or amplifying neural progenitors (ANP), using cell morphology and GFP and GFAP expression to distinguish between the classes of progenitors as described (Encinas and Enikolopov, 2008; Park and Enikolopov, 2010; Encinas et al., 2011a,b; Park et al., 2013).

### Statistics

One-way ANOVA followed by *post-hoc* comparison using Bonferroni test was performed to reveal the effects of factor “lines” on the parameters of aggressive behavior. *X*^2^ was used for comparing the percentages of males demonstrating rotations, aggressive grooming and threats. For analysis of the parameters of agonistic behaviors of 21 day winners before and after 14 days of a no-fight period (Win21 and Win21-D, respectively), Wilcoxon matched pairs test was used. For the plus maze experiments *t*-test was used. For analysis of the partition behavior, ANOVA for repeated measures with comparison using paired *t*-test was used to analyze winners' reaction to familiar or unfamiliar partners in the neighboring compartment of a common cage. Statistical analysis of c-fos expression in different regions of amygdala was performed using Two-way ANOVA for factor “groups” (controls, Win21), factor “regions” (basolateral, medial, and central) and factor “interactions” under consideration followed by the comparison of the groups using the *t*-test. One-way ANOVA of the data with factor “experience” (controls, Win10, Win21) and factor “deprivation” (controls, Win21, and Win21-D) followed by the *post-hoc* comparison of the groups using Bonferroni correction was applied to compare the numbers of BrdU- and c-fos-positive cells in all groups. The data are reported as mean ± SEM. The statistical significance was set at *P* = 0.05. For behavioral experiments each experimental group contained 6–13 animals, and 5–12 animals were used for immunocytochemical experiments.

## Results

### Parameters of aggressive behavior

We exposed adult males of several wild type and reporter strains to conditions eliciting agonistic interactions and determined key parameters of aggressive behavior in the animals showing positive fighting history (i.e., scored as winners). Animals of all examined groups (Nestin-GFP homozygotes, Nestin-GFP heterozygotes, wild type C57BL/6J, and wild type C57BL/Icg) displayed aggression after multiple encounters, although the fraction of males demonstrating aggression during the 21-day period differed for these groups (from ~70% for C57BL/6J and Nestin-GFP to ~90% for C57BL/Icg mice). We next used One-way and repeated measurements ANOVA to investigate features of aggressive behavior in the winners of the four lines.

In the winning mice, One-way ANOVA revealed the influence of the factor “line” (C57BL/Icg, C57BL/6J, Nestin-GFP, or Nestin-GFPhet) on the total time of attacks [*F*_(3, 32)_ = 6.79; *P* < 0.001]; total time of aggressive grooming [*F*_(3, 32)_ = 4.56; *P* < 0.009]; number [*F*_(3, 32)_ = 6.27; *P* < 0.002] and total time of digging behavior [*F*_(3, 32)_ = 19.14; *P* < 0.0001]; the total time of hostile behavior [*F*_(2, 27)_ = 6.60; *P* < 0.0001]; and number of threats [*F*_(3, 32)_ = 3.50; *P* < 0.027; Table 1]. In Nestin-GFPhet mice the total time of attacks was significantly higher than in C57BL/6J (*P* < 0.006), Nestin-GFP (*P* < 0.002), or C57BL/Icg (*P* < 0.002) mice, the total time of hostile behavior was significantly higher than in Nestin-GFP (*P* < 0.001) and the total time of digging behavior was significantly less than in C57BL/6J (*P* < 0.001; all values obtained after Bonferroni correction). Additionally, Nestin-GFP mice differed in comparison with C57BL/6J and C57BL/Icg mice in the number of diggings (*P* < 0.003 and *P* < 0.005, respectively); with C57BL/6J mice in the total time of diggings—(*P* < 0.001); and with C57BL/Icg mice in the total time of aggressive grooming (*P* < 0.029). In Nestin-GFP mice total time of hostile behavior was significantly less than in C57BL/6J (*P* < 0.014) and C57BL/Icg (*P* < 0.039) mice. In C57BL/6J mice total time of digging behavior was significantly higher than in C57BL/Icg mice (*P* < 0.001).

**Table 1:** **Behavior of C57BL/Icg, C57BL/6J, Nestin-GFP, and Nestin-GFPhet winners in agonistic interactions**.

**Behavioral parameters**	C57BL/Icg (^*^)	**C57BL/6J (^+^)**	**Nestin-GFP (^&^)**	**Nestin-GFPhet**
Latency of attacks, s	180.8 ± 59.7	233.7 ± 51.7	295.9 ± 75.7	130.4 ± 52.7
Attacks, *N*	5.3 ± 1.0	4.3 ± 1.3	2.6 ± 0.8	7.5 ± 1.5
Attacks, s	34.2 ± 6.5	38.9 ± 17.3	25.8 ± 12.2	125.8 ± 30.3^**^^++^^&^
Diggings, *N*	21.3 ± 2.8	22.3 ± 1.3	8.3 ± 1.8^++^^**^	15.3 ± 4.0
Diggings, s	44.1 ± 4.3	94.0 ± 7.1^***^	26.8 ± 4.8^+++^	43.7 ± 12.8^+++^
Hostile behaviors, s	125.4 ± 12.3	138.1 ± 19.9	54.4 ± 12.6^*^^+^	175.7 ± 29.9^&&^
Rotations, %	67	100	87.5	83
Aggressive grooming, %	50	20	12.5	50
Threats, % males	50	0^#^	0^#^	50
Animals, *N*	12	10	8	6

* Refers to comparisons with C57BL/Icg,

+ refers to C57BL/6J,

& refers to Nestin-GFP, and

^#^refers to C57BL/Icg and Nestin-GFPhet; i.e.,

* P < 0.05;

** P < 0.01;

*** P < 0.001 vs. C57BL/Icg;

+ P < 0.05;

++ P < 0.01;

+++ P < 0.001 vs. C57BL/6J;

& P < 0.01;

&& < 0.001 vs. Nestin-GFP;

# *P < 0.05 vs. C57BL/Icg and Nestin-GFPhet*.

In addition, percentage of C57BL/Icg and Nestin-GFPhet males demonstrating threats was significantly higher than for C57BL/6J or Nestin-GFP mice (*P* < 0.012 for C57BL/Icg vs. C57BL/6J comparison; *P* < 0.042 for C57BL/Icg vs. Nestin-GFP; *P* < 0.036 for Nestin-GFPhet vs. C57BL/6J, and *P* = 0.05 for Nestin-GFPhet vs. Nestin-GFP; revealed by the *X*^2^-test). Finally, percentage of male mice demonstrating episodes of aggressive grooming and rotations did not differ significantly between the groups (70–100% of male mice in each group displaying rotations; *P* > 0.05; Table 1).

### Behavior in the partition test

When winners of different groups and the respective control were exposed to the partition test (which reflects aggressive motivation) and their behavioral reactions to familiar and unfamiliar male partners were scored, One-way ANOVA for repeated measures revealed significant differences: for the Nestin-NGFP the influence of factor “status” [control, winners; *F*_(1, 13)_ = 5.31; *P* < 0.038], factor “partner” [familiar-unfamiliar; *F*_(1, 13)_ = 4.77; *P* < 0.048], and interactions [*F*_(1, 13)_ = 13.79; *P* < 0.003]; for C57BL/6J the influence of factor “partner” [*F*_(1, 18)_ = 27.14; *P* < 0.001] and tendency for interactions [*F*_(1, 18)_ = 3.10; *P* < 0.095]; for C57BL/Icg the influence of factor “partner” [*F*_(1, 23)_ = 42.87; *P* < 0.0001] and interactions [*F*_(1, 18)_ = 12.09; *P* < 0.002]; for Nestin-GFPhet the influence of factor “status” [*F*_(1, 11)_ = 18.06; *P* < 0.001] and factor “partner” [*F*_(1, 11)_ = 5.68; *P* < 0.036].

**Figure 1:**
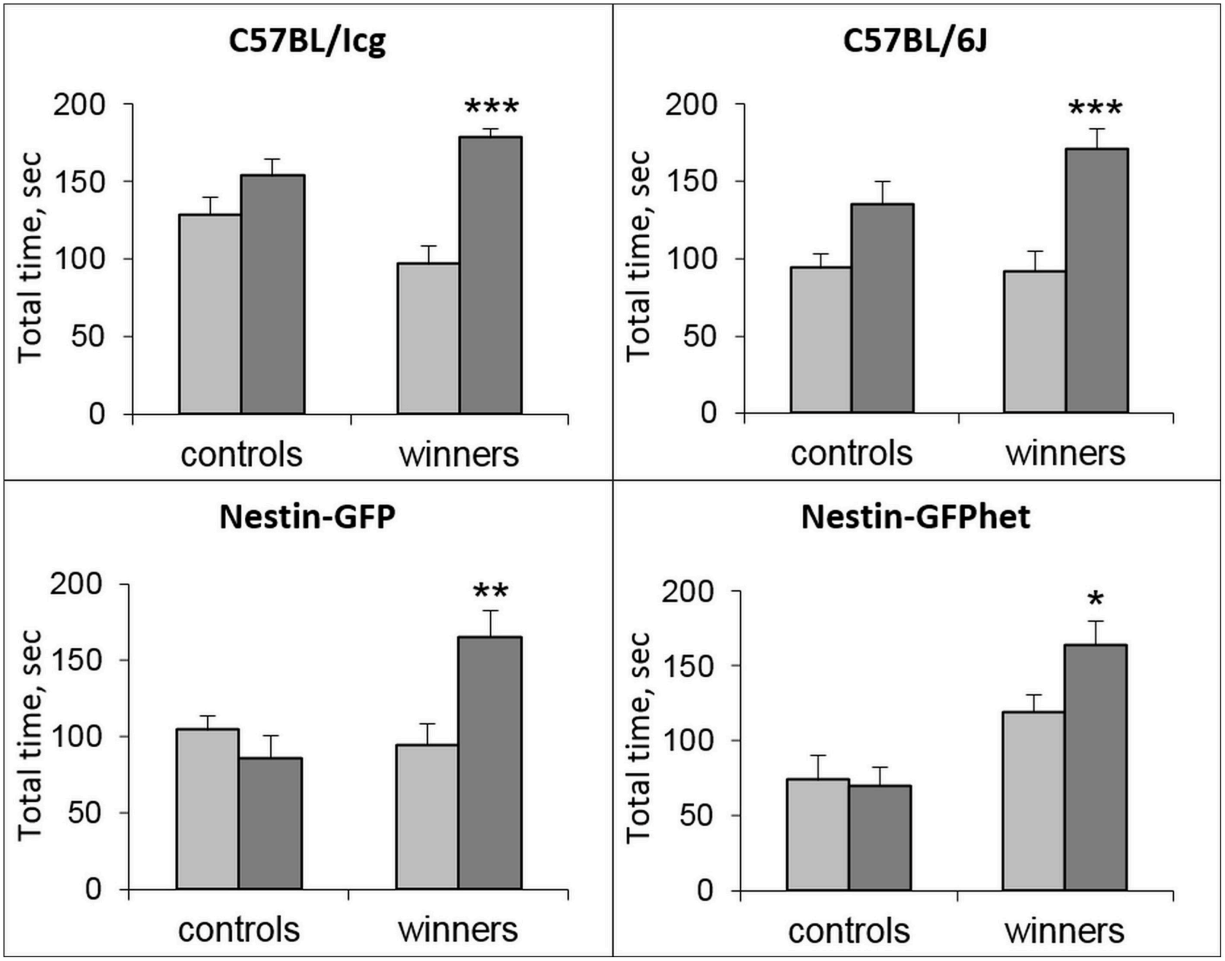
**Partition test for male mice of different lines**. Total time spent near the partition by the male mice of four lines (see captions) in the control and Win-21 groups as reaction to the familiar (light bars) and unfamiliar (dark bars) partners in the neighboring compartment of the common cage. ^*^P < 0.05, ^**^P < 0.01, ^***^P < 0.001; paired *t*-test for unfamiliar vs. familiar partner.

In all strains, significant differences were revealed in the total time spent near the partition, considered as behavioral reaction of the winners to unfamiliar partners in comparison with familiar partners: C57BL/Icg (*t* = 6.27; *P* < 0.001), C57BL/6J (*t* = 5.58; *P* < 0.001), Nestin-GFP (*t* = 5.94; *P* < 0.003), and Nestin-GFPhet (*t* = 2.96; *P* < 0.025) lines (Figure 1).

### Behavior of the winners in the plus-maze test

Positive fighting experience can heighten anxiety in mice (Kudryavtseva et al., 2002); therefore, we examined the winners of different lines in the elevated plus-maze test. We found that repeated experience of aggression increased several parameters of anxiety-driven behavior in the winners of the C57BL/6J, Nestin-GFP, and Nestin-GFPhet lines (Table 2). While the overall tendency was similar for these lines for most parameters, results showed a significantly increased number of closed-arm entries (*t* = 2.19, *P* < 0.049) and increased number of peepings (*t* = 2.34, *P* < 0.037) for the winners of the Nestin-GFP line as compared to the control animals; a significantly increased number of closed-arm entries (*t* = 2.14; *P* = 0.05), a decreased number of open-arm entries (*t* = 2.15; *P* = 0.05) and decreased time spent in open arms (*t* = 2.28; *P* < 0.040) for the Nestin-GFPhet winners; and decreased time spent in the central platform (*t* = 2.67; *P* < 0.017) for the C57BL/6J winners. Taken together, our experiments demonstrate that repeated positive winning experience enhances aggressive behavior in the winning animals of all tested lines, but also increases their anxiety.

**Table 2:** **Behavior in the elevated plus-maze test of the Nestin-GFP, C57BL/6J, and Nestin-GFPhet winners after 21 days of agonistic interactions**.

**Behavioral parameters**	**Nestin-GFP**		**C57BL/6J**		**Nestin-GFPhet**	
	**Control**	**Winners**	**Control**	**Winners**	**Control**	**Winners**
Closed arm entries, %	36.4 ± 4.2	47.6 ± 1.5^*^	39.4 ± 2.1	39.4 ± 2.6	28.9 ± 5.6	43.0 ± 2.8^*^
Closed arm time, %	56.5 ± 8.6	77.7 ± 3.3	59.0 ± 7.9	72.1 ± 3.7	44.5 ± 10.7	70.3 ± 6.0
Central platform entries, %	50.5 ± 0.3	50.0 ± 0.0	50.5 ± 0.3	50.0 ± 0.0	50.5 ± 0.3	50.3 ± 0.2
Central platform time	26.2 ± 4.2	19.8 ± 2.0	35.1 ± 6.7	18.3 ± 1.8^*^	16.5 ± 2.0	17.9 ± 2.7
Open arm entries, %	13.1 ± 4.2	2.4 ± 1.5	10.1 ± 1.9	10.6 ± 2.6	20.6 ± 5.5	6.7 ± 2.8^*^
Open arm time, %	17.3 ± 7.9	2.6 ± 1.7	5.8 ± 1.4	9.3 ± 2.7	39.0 ± 10.5	11.8 ± 4.0^*^
Total entries	32.8 ± 3.3	34.0 ± 3.3	25.5 ± 2.9	24.0 ± 3.2	34.8 ± 3.9	44.3 ± 2.2
Passages, *N*	8.4 ± 1.9	12.5 ± 1.1	6.5 ± 1.5	5.2 ± 1.0	8.8 ± 2.7	15.0 ± 1.8
Peepings, *N*	4.6 ± 1.1	8.8 ± 1.4^*^	5.4 ± 1.0	7.0 ± 1.7	1.6 ± 0.6	3.7 ± 1.2
Head-dips, *N*	11.8 ± 3.5	7.3 ± 1.6	6.3 ± 0.8	8.2 ± 1.7	15.0 ± 3.3	9.8 ± 2.5
Animals, *N*	8	6	8	10	8	7

* *P < 0.05 vs. respective controls, t-test*.

### Aggressive behavior of winner animals after fight deprivation

We next asked whether a no-fight period induces additional behavioral changes in mice with prolonged winning experience. We compared winning animals after 21 days of agonistic interactions (Win21) and winning animals which, after 21 days of agonistic interactions, were deprived of fighting experience for 14 days (Win21-D; these series of experiments were performed with the C57BL/Icg male mice). After the deprivation period, we found significant changes in several parameters of aggressive behavior, such as a decrease in the latency of the first attack (*Z* = 1.96; *P* < 0.05), an increase in total attacking time (*Z* = 1.96; *P* < 0.05), and an increase in the meantime of one attack (*Z* = 2.31; *P* < 0.021; Table 3). Together, these results indicate that aggressive behavior is further augmented in mice that were deprived of fighting after a prolonged series of winning.

**Table 3:** **Behavior of Win21 C57BL/Icg male mice in the agonistic interaction test before and after deprivation**.

**Behavioral parameters**	**Before deprivation**	**After deprivation**
Latency of attacks, s	113.3 ± 53.5	41.9 ± 14.9^*^
Attacks, s	26.9 ± 10.7	60.3 ± 14.0^*^
Attacks, *N*	6.5 ± 1.8	9.8 ± 1.5
Attacks, *T*/*N*	3.5 ± 0.5	6.6 ± 1.1^*^
Diggings, s	22.1 ± 4.5	21.1 ± 3.9
Diggings, *N*	14.8 ± 2.8	10.9 ± 1.7
Hostile behavior, s	73.0 ± 9.2	99.1 ± 16.3
Rotations, *N*	3.1 ± 0.7	6.0 ± 1.6
Threats, % males	12	50
Animals, *N*	8	8

* *P < 0.05 vs. males before fighting deprivation, Wilcoxon matched pairs test*.

### Division of hippocampal progenitors

**Figure 2:**
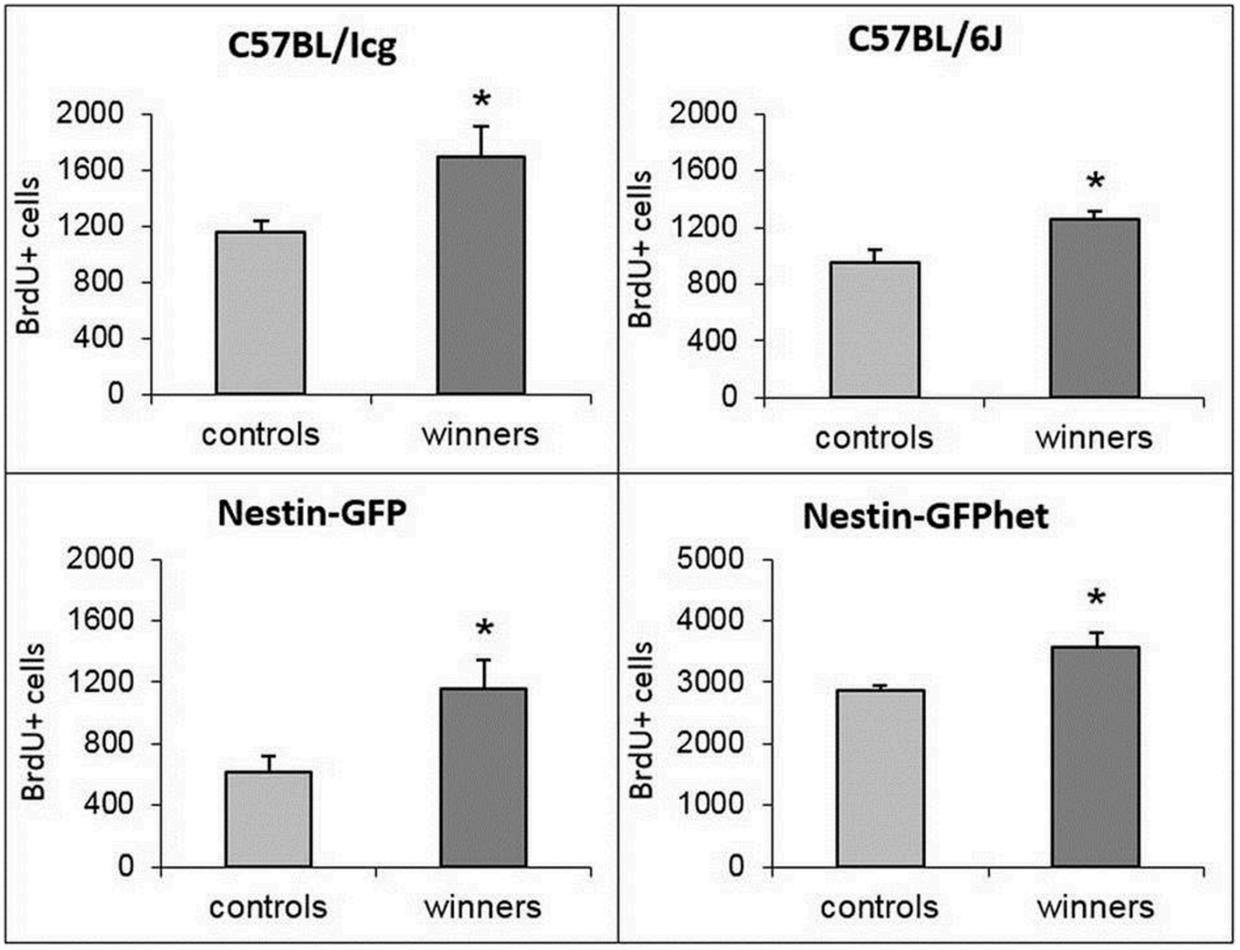
**BrdU-positive cells in the SGZ of the DG of control and winning males of four lines (captions)**. ^*^P < 0.05, *t*-test.

In male rodents, DG of the hippocampus, a site of ongoing neurogenesis in the adult brain, responds to social defeat stress by a decrease in production of new neurons, whereas an elevated social status correlates with increased level of cell division in the DG (Kozorovitskiy and Gould, 2004; Veenema et al., 2004, 2007; Yap et al., 2006; Lagace et al., 2010; Samuels and Hen, 2011; Cameron and Glover, 2015). Therefore, we compared division of stem and progenitor cells in the DG of control and winner groups of animals by labeling dividing cells with thymidine analog BrdU. For all four analyzed lines we found a significant increase in the number of BrdU cells in the DG of winners (*t* = 2.81, *P* < 0.018 for C57BL/Icg; *t* = 2.92, *P* < 0.015 for C57BL/6J; *t* = 2.583, *P* < 0.033 for Nestin-GFP, and *t* = 2.34, *P* < 0.036 for Nestin-GFPhet), ranging from 24% in Nestin-GFPhet to 90% in Nestin-GFP animals (Figure 2).

**Figure 3:**
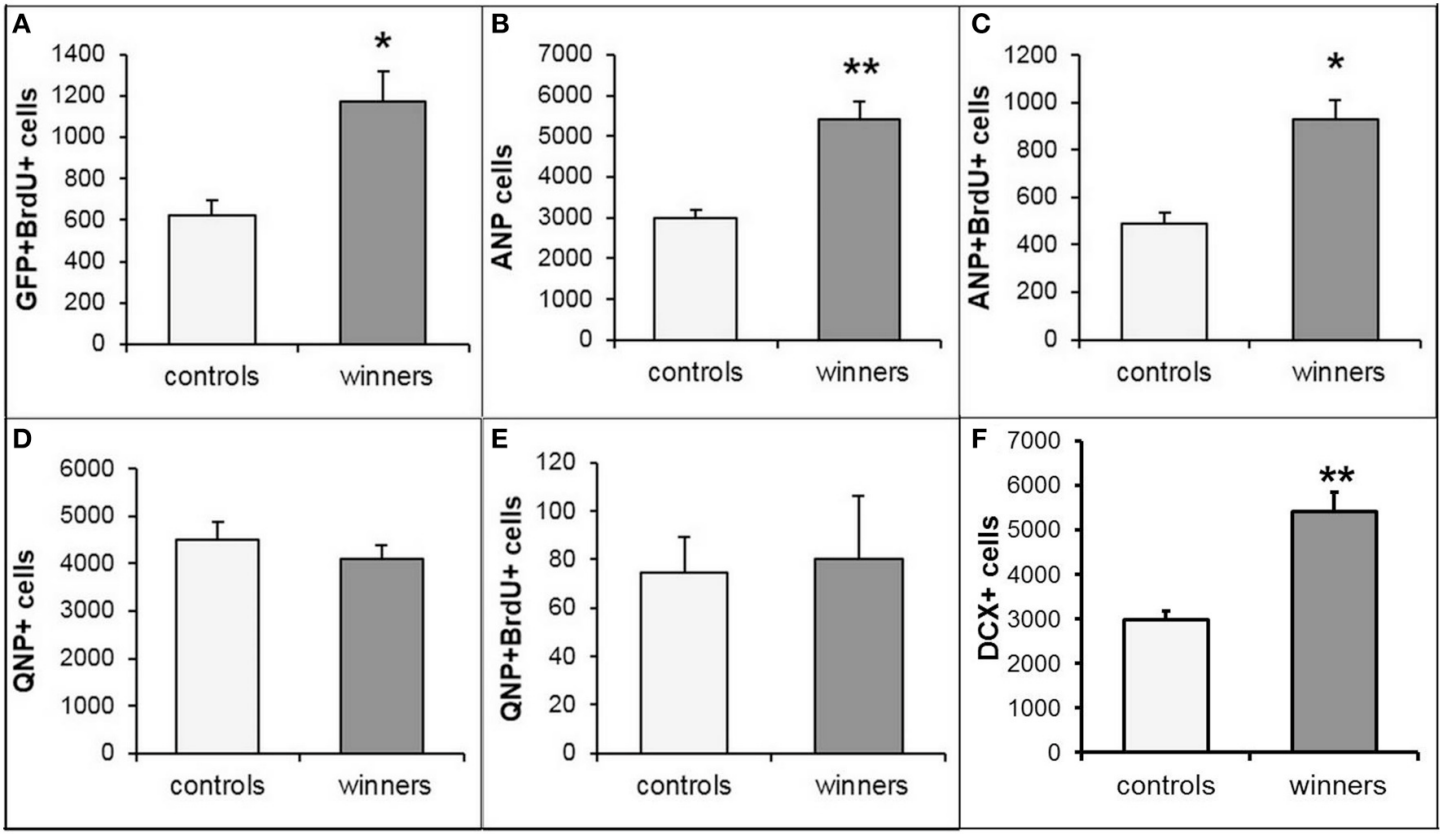
**Subclasses of progenitor cells in the SGZ of the DG of Nestin-GFP male mice**. Numbers of dividing GFP^+^BrdU^+^ progenitor cells **(A)**, ANP cells **(B)**, dividing ANP cells **(C)**, QNP cells **(D)**, dividing QNP cells **(E)**, and Dcx^+^ young neurons **(F)**. ^*^P < 0.05, ^**^P < 0.01; *t*-test.

Incorporation of thymidine analogs, while marking cells undergoing DNA synthesis, does not distinguish between neuronal progenitors and other dividing cells types (e.g., oligodendrocyte progenitors, microglia, pericytes, or endothelial cells) or between different classes of neuronal progenitors (e.g., stem cells and their amplifying progeny). Therefore, we further analyzed mice of the reporter Nestin-GFP line in which various classes of neuronal progenitors can be distinguished from other classes of progenitors or other cell types based on the expression of the reporter, morphology, and additional markers (Mignone et al., 2004; Enikolopov et al., 2015). We analyzed the number of GFP-positive cells in the subgranular zone (SGZ) that were labeled with BrdU and found that the number of GFP^+^BrdU^+^ cells was increased, indicating that the increase in dividing cells was driven by neuronal progenitors. The majority of GFP^+^BrdU^+^ cells in adult mice of this reporter line corresponds to amplifying neural progenitors (ANP), whereas a smaller fraction corresponds to the quiescent neural stem cell population (QNP; Encinas et al., 2011b). These two cell types can be distinguished by their morphology (e.g., by the radial glia-like shape of quiescent stem cells) aided by marker expression (GFAP expression in the stem cells, but not in their amplifying progeny). We found an increase in the overall number of ANPs and in the number of BrdU-positive ANPs, but not in the number of QNPs or dividing QNPs (Figure 3). This suggests that prolonged winning experience in agonistic interactions does not change the number of the hippocampal stem cells, but increases the number of their rapidly dividing progeny.

Since our social conflict protocol covered 3 weeks, positive fighting experience may have also impacted the population of young newly born hippocampal neurons. Bulk of young differentiating neurons in the DG express doublecortin (Dcx); therefore, we determined the changes in the number of Dcx-positive cells in the SGZ and the adjacent region of the granule cell layer in the same specimens as those used to quantify progenitors and dividing progenitors. We found an increased number of Dcx-positive young neurons in the winners group of Nestin-GFP reporter animals (Figure 3F). This increase is comparable to the increases observed with BrdU-, GFP/BrdU-, ANP-, and ANP/BrdU-positive cells in the same specimens. These results indicate that prolonged positive fighting experience increases the number of young neurons in the DG, suggesting that the increase in proliferation of progenitor cells is translated into augmented production of young neurons.

### Division of neuronal progenitors and neuronal activity in the hippocampus of aggressive males

**Figure 4:**
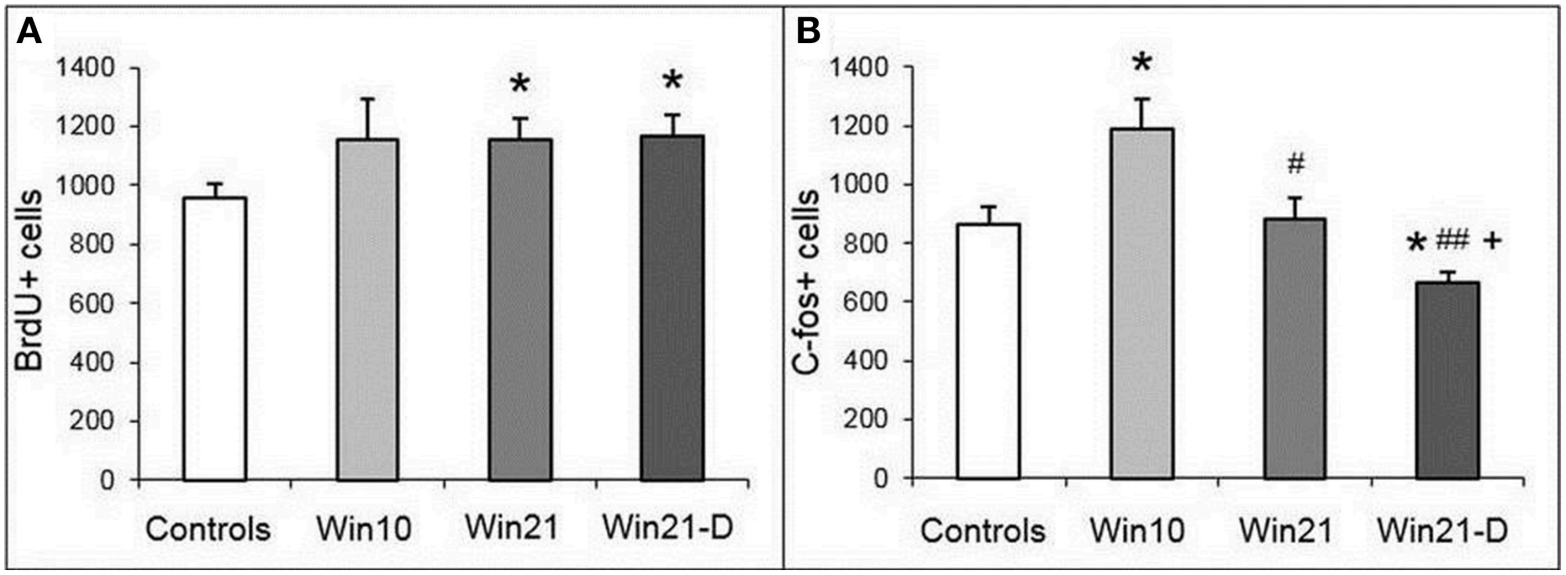
**BrdU-positive cells (A) and c-fos labeled cells (B) in the DG of male mice with different fighting experience**. C57BL/Icg male mice: Win10, 10 day winners; Win21, 21 day winners; Win21-D, 21day winners after 2 weeks of fighting deprivation. ^*^P < 0.05 vs. the controls; ^#^*P* < 0.05 and ^*##*^*P* < 0.01 vs. Win10; ^+^*P* < 0.05 vs. Win21; *t*-test.

We next asked whether fight deprivation affects cell division in the DG. In these experiments we compared, in addition to the controls, the Win 10, Win21, and Win21-D groups described above. One-way ANOVA revealed influence of factor “deprivation” [Control, Win21, Win21-D; *F*_(2, 36)_ = 3.91; *P* < 0.029] on the number of BrdU-positive cells in DG of hippocampus. *t*-test revealed significant differences between the control and Win21 groups (*t* = 2.41; *P* < 0.022) and the control and Win21-D groups (*t* = 2.48; *P* < 0.021). Thus, the number of BrdU-positive cells in the SGZ was increased in the animals with 21 days of winning experience, with or without subsequent deprivation (Figure 4A).

We then asked whether neuronal activity is also altered in mice with or without fight deprivation by relying on c-fos immunoreactivity as a surrogate marker of neuronal activity. One-way ANOVA revealed influence of factor “experience” [Control, Win10, Win21; *F*_(2, 14)_ = 5.141; *P* < 0.021] and factor “deprivation” [Control, Win21, Win21-D; *F*_(2, 13)_ = 3.91; *P* < 0.047] on the number of c-fos-positive cells in DG of the hippocampus. *t*-Test revealed significant differences between the controls and Win10 (*t* = 2.74; *p* < 0.021); controls and Win21-D (*t* = 2.65; *p* < 0.026); Win10 and Win21 (*t* = 2.34; *p* < 0.044); Win10 and Win21-D (*t* = 4.48; *p* < 0.002); and Win20 and Win21-D groups (*t* = 2.64; *p* < 0.030). Thus, the number of c-fos-positive cells was increased in the Win10 group compared to the control, was decreased in the Win21 compared to the Win10 group, and was decreased in the Win21-D group compared to Win10, Win21, and control groups (Figure 4B).

### Neuronal activity in the amygdala of aggressive males

**Figure 5:**
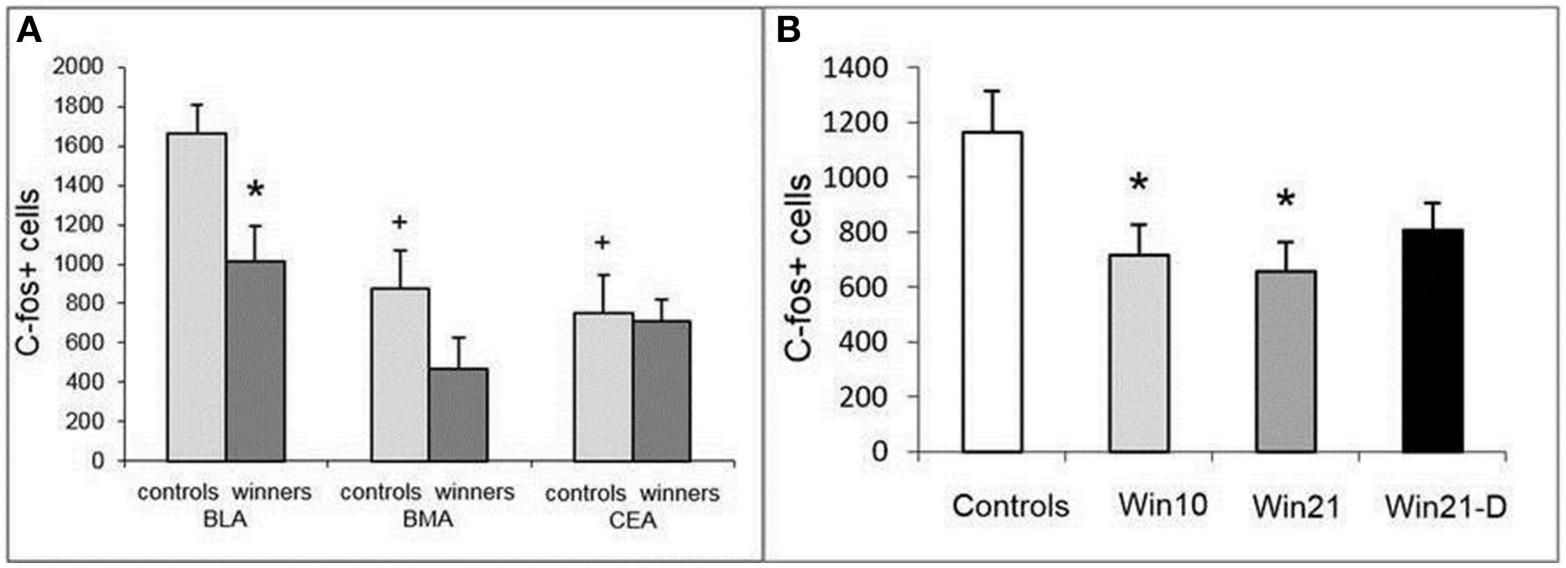
**c-fos-Positive cells in the amygdala of male mice after 21 days of agonistic interactions**. **(A)** c-fos cells in different regions of the amygdala of control and winning C57BL/Icg male mice: BLA, basolateral amygdala; BMA, basomedial amygdala; CEA, central amygdala. **(B)** c-fos cells in the basolateral amygdala (BLA) of C57BL/Icg male mice with different fighting experience: Win10, 10 day winners; Win21, 21 day winners; Win21-D, 21 day winners after 2 weeks of fighting deprivation. ^*^P < 0.05 vs. the controls, ^+^*P* < 0.01 vs. controls in BLA; *t*-test.

Having detected changes in neuronal activity in the SGZ of the DG, we asked whether it is also altered in the amygdala. We first compared the number of c-fos cells in selected regions of the amygdala (BLA, BMA, and CeA) in control and Win21 males (Figure 5A). Two-way ANOVA with *post-hoc t*-test revealed the influence of factor “area” [*F*_(2, 36)_ = 9.194; *P* < 0.001] and factor “groups” [control, winners; *F*_(1, 36)_ = 6.809; *P* < 0.013]. *t*-Test revealed a significant decrease in the number of c-fos-positive cells in the BLA of the Win21 mice compared to the control animals (*t* = 2.84; *P* < 0.015). There was also a significant difference between the amygdala regions in the control animals (a smaller number of c-fos cells in the BMA (*t* = 3.31; *P* < 0.005) or CeA (*t* = 3.78; *P* < 0.002) than in BLA; factor “regions”), but not in the Win21 animals (factor “interactions”).

After establishing the difference in activated cells in the BLA of the winner males, we compared the number of c-fos-expressing cells in the BLA of control, Win10, Win21, and Win21-D groups (Figure 5B). One-way ANOVA with *post-hoc t*-test revealed a significant influence of the factor “deprivation” [Controls, Win21, Win21-D; *F*_(2, 15)_ = 4.73; *P* < 0.025] and factor “experience” [Controls, Win10, Win21; *F*_(2, 15)_ = 5.001; *P* < 0.021] on the number of c-fos-positive cells. *t*-Test revealed a significant decrease in the number of c-fos-positive cells in Win10 (t = 2.41; P < 0.037) and Win21 (*t* = 2.78; *P* < 0.019) groups in comparison to the controls. Together, these results indicate that positive fighting experience affects the number of activated neurons in the BLA.

## Discussion

Using several mouse lines, we demonstrate that prolonged positive fighting experience increases proliferation of neuronal progenitors and neurogenesis in the DG of adult males. This increase is preserved after a 2 week period of fight deprivation. Augmented neurogenesis in the DG is contrasted by a decrease in activation of the DG neurons (following an initial increase). Positive fighting experience is also accompanied by a change in neuronal activity in the amygdala. Together, our results point to a close link between a series of winning in a social conflict paradigm and changes in neuronal plasticity in key limbic regions of the adult brain.

After 21 days of agonistic interactions accompanied by positive fighting experience, winners of all four examined mouse lines demonstrated aggressive behavior in agonistic interaction test, attesting to the robustness of the experimental approach (even though the dynamics of getting engaged in agonistic interactions were different among the examined lines). These animal groups did not differ in expression of direct aggression, e.g., in the latency of the first attacks and the number of the attack; however, overall duration of attacks was significantly higher in Nestin-GFPhet (Table 1). Mouse lines also did not differ in the proportion of winners demonstrating rotations (which can be considered as a stereotypic reaction), threats, or aggressive grooming (a ritualized form of aggressive behavior). However, the lines differed in the parameters of indirect aggression, such as the number of diggings on the territory of the defeated animal or the overall duration of the digging episodes; they also differed in the total duration of hostile behavior by the winner, including direct, indirect, and ritualized forms of aggression (higher in C57BL/6J and C57BL/Icg than in Nestin-GFP males). Furthermore, winners of C57BL/Icg and Nestin-GFPhet, but not C 57BL/6J or Nestin-GFP, lines demonstrated threats along with direct aggression (attacks). Interestingly, while Nestin-GFPhet males were less prone to demonstrating aggression upon first interactions, Nestin-GFPhet winners were remarkably more aggressive overall as judged by the total time of attacks. Nestin-GFP winners were least aggressive in agonistic interactions as estimated by the total time of hostile behavior.

Notably, in the partition test winners of all examined lines spent more total time near the partition in reaction to unfamiliar partners than to familiar ones (Figure 1). Such increase (which correlates with the level of aggressiveness if the partition is removed) reflects an increase in the level of aggressive motivation (Kudryavtseva, 2003). In addition, winners of all groups demonstrated increased levels of anxiety (Table 2), confirming the data on elevated anxiety in the C57BL/Icg winners (Kudryavtseva et al., 2002). Together, these results show that positive fighting experience reliably induces changes in social behaviors and psychoemotional state, manifested as increased aggressive motivation and heightened anxiety.

Our results show that repeated experience of aggression elevates division of neuronal progenitors in the SGZ of the hippocampus; this was observed in all strains analyzed (albeit to a different degree in different strains). Importantly, production of Dcx-positive young differentiating neurons was also elevated, suggesting that the initial increase in proliferation of progenitors is later manifested as increased neurogenesis. Notably, 14 days of fight deprivation did not decrease the augmented cell division to the control levels; this may parallel our observations that anxiety levels, increased in the winners, are not decreased to control levels after fight deprivation (Smagin et al., 2010).

Our results also show that the main contribution to the increase in cell division is provided by the rapidly dividing progeny of neural stem cells, but not by the stem cell population itself. This resembles the action of the majority of the pro-neurogenic treatments and compounds, such as deep brain stimulation (Encinas et al., 2011a), fluoxetine (Encinas et al., 2006), or running (Kronenberg et al., 2003; Hodge et al., 2008), highlighting transit amplifying progenitor population as a common target of diverse pro-neurogenic stimuli.

Our results on increased neurogenesis in the aggressive males match the results obtained with mouse strains selected for elevated level of aggressive behavior (Veenema et al., 2004, 2007) and rats with a dominant position in the hierarchy (Kozorovitskiy and Gould, 2004). Remarkably, the direction of these changes is opposite to the changes in hippocampal neurogenesis observed in mice and rats with negative experience in social conflicts: chronic social defeat in daily agonistic interactions is associated with depression- and anxiety-like state and results in decreased cell division and neurogenesis in the DG (Buwalda et al., 2005; Ferragud et al., 2010; Lagace et al., 2010; Van Bokhoven et al., 2011). This complementarity may reflect a balance between neuromodulatory systems of the brain, e.g., dopaminergic and serotonergic, with activation of the former and inhibition of the latter noted in males with positive fighting experience (Kudriavtseva and Bakshtanovskaia, 1991; Filipenko et al., 2001; Miczek et al., 2007; Ginsberg et al., 2011; Comai et al., 2012). More generally, these observations may be relevant to the rewarding features of aggression in animals and humans, with positive reinforcement predisposing for further aggressive behavior.

Anxiety is usually associated with decreased progenitor proliferation and neurogenesis (Lagace et al., 2010; Samuels and Hen, 2011; Cameron and Glover, 2015); however, our data show that the winner males show both augmented proliferation and neurogenesis and increased anxiety in the elevated plus maze test (although increase in anxiety is much more pronounced in the defeated partner; data not shown). There can be a number of possible explanations of this intriguing observation: for instance, it is conceivable that in the social conflict setting that we have applied, the daily stress of agonistic interactions that is shared by both partners elevates anxiety in the elevated plus maze test (albeit to a different degree), whereas changes in proliferation and neurogenesis are more related to the repeated fighting experience (positive in winner males and negative in defeated males). It is also possible that on the common background of the social stress of agonistic interactions, the pronounced activation of the dopaminergic system in the brain (Filipenko et al., 2001) demonstrated for this model, has a particular effect on hippocampal neurogenesis. It is also conceivable that fine features of anxiety are not identical in the winners and losers and that anxiety in winners, as measured in the elevated plus maze test, is not directly related to the changes in hippocampal neurogenesis. In addition, it is possible that the differences in anxiety vs. neurogenesis levels are related to some threshold effect that anxiety has on neurogenesis and vice versa. More generally, the connection between anxiety and neurogenesis may be clarified if the underlying effect of the activated hypothalamic-pituitary-adrenal (HPA) axis is investigated in our paradigm of a prolonged social conflict.

There are numerous indications of a link between hippocampal neurogenesis and amygdalar activity (Cameron and Glover, 2015), neurogenesis being particularly sensitive to emotional inputs from the BLA. Our results show that the number of c-fos-expressing cells, considered a reporter of neuronal activity, is lower in the BLA (but not in the BMA or CeA) of the winners than in the BLA (or corresponding regions) of control animals. Furthermore, this number increases after 10 days of agonistic interactions but decreases after 21 days of fighting and, in particular, after fight deprivation. This suggests that BLA may be involved in aggressive behavior or associated behaviors in males with prolonged (21 days) positive fighting experience.

These results are consistent both with the notion of functional heterogeneity of the amygdala and the role of the BLA in plasticity of the stimulus-value association (Knapska et al., 2007). They are also compatible with the notion of the amygdalar activity reflecting the novelty of the stimulus, sometimes irrespective of the valence of the stimulus. This may explain the differences in the reports of c-fos activity detected in various nuclei of amygdala (and other brain regions) in mice, rats, and hamsters after a single or short repeated experience of social defeat (Kollack-Walker et al., 1997; Martinez et al., 1998; Fekete et al., 2009; Pan et al., 2010; Wohleb et al., 2011; Morrison et al., 2012), but also in the brain of dominant and aggressive animals, or even in the amygdalar nuclei of both subordinate and dominant animals (Kollack-Walker et al., 1997; Pan et al., 2010; Morrison et al., 2012).

A potentially interesting connection may exist between amygdalar activity in the winner animals and development of psychopathological features of social behavior observed after prolonged periods of aggressive behavior. The amygdala is thought to be involved in various psychopathologies, including personality disorders, attention deficit/hyperactivity disorder, major depressive disorder, and anxiety disorders (Baron-Cohen et al., 2000; Plessen et al., 2006; Etkin and Wager, 2007; Goossens et al., 2007; Silbersweig et al., 2007; Garrett and Chang, 2008; Monk, 2008). The amygdala is linked to regulation of emotions and therefore is expected to play a role in both aggression and social withdrawal, probably through modulation of fear-induced behaviors (Lopez et al., 2004).

Changes in amygdalar activity in winners may point to another potentially intriguing connection, with social pathology in autism. We note the triad of autism spectrum disorder symptoms—impairment of sociability, low communication, and repetitive behaviors, in males with prolonged aggressive experience. Such animals show impaired communication and impaired behavior; disturbances in social recognition; increased locomotion, hyperactivity, and chaotic activity without attention toward the partner; decreased exploration; and various forms of stereotypic repeated behaviors (jumps, back circles, turning movements around the body axis; high muscular tension and rigid tail; Kudryavtseva et al., 2014). These changes are consistent with the amygdalar dysfunction being proposed as a critical component in social impairment in autism spectrum disorders (Baron-Cohen et al., 2000; Neuhaus et al., 2010), with functional magnetic resonance imaging demonstrating that patients with autism spectrum disorders show hypoactivation in the amygdala in response to social and non-social rewards and have atypical and significantly reduced neural functional connectivity within the amygdala and between the amygdala and other “social” brain regions (Ebisch et al., 2011; Jou et al., 2011; Ecker et al., 2012; Kohls et al., 2013; von dem Hagen et al., 2013). Impaired social behavior characterizes many animal models of the disorder, supporting the notion that analysis of pathologies observed in mice with prolonged fighting experience may provide valuable insights into the deficits in social interactions observed in humans with autism spectrum disorder.

### Conflict of interest statement

The authors declare that the research was conducted in the absence of any commercial or financial relationships that could be construed as a potential conflict of interest.
